# Preclinical evaluation of the first intravenous small molecule MDM2 antagonist alone and in combination with temozolomide in neuroblastoma

**DOI:** 10.1002/ijc.32058

**Published:** 2019-01-09

**Authors:** Lindi Chen, Fabio Pastorino, Philip Berry, Jennifer Bonner, Calum Kirk, Katrina M. Wood, Huw D. Thomas, Yan Zhao, Antonio Daga, Gareth J. Veal, John Lunec, David R. Newell, Mirco Ponzoni, Deborah A. Tweddle

**Affiliations:** ^1^ Wolfson Childhood Cancer Research Centre Northern Institute for Cancer Research, Newcastle University Newcastle upon Tyne United Kingdom; ^2^ Laboratory of Experimental Therapy in Oncology IRCCS Istituto Giannina Gaslini Genoa Italy; ^3^ Northern Institute for Cancer Research, Newcastle University Newcastle upon Tyne United Kingdom; ^4^ Department of Cellular Pathology Royal Victoria Infirmary Newcastle upon Tyne United Kingdom; ^5^ Oncologia Cellulare IRCCS Ospedale Policlinico San Martino Genoa Italy

**Keywords:** neuroblastoma, MDM2 antagonists, idasanutlin (RG7388), RO6839921 (RG7775), temozolomide

## Abstract

High‐risk neuroblastoma, a predominantly *TP53* wild‐type (wt) tumour, is incurable in >50% patients supporting the use of MDM2 antagonists as novel therapeutics. Idasanutlin (RG7388) shows *in vitro* synergy with chemotherapies used to treat neuroblastoma. This is the first study to evaluate the *in vivo* efficacy of the intravenous idasanutlin prodrug, RO6839921 (RG7775), both alone and in combination with temozolomide in *TP53* wt orthotopic neuroblastoma models. Detection of active idasanutlin using liquid chromatography‐mass spectrometry and p53 pathway activation by ELISA assays and Western analysis showed peak plasma levels 1 h post‐treatment with maximal p53 pathway activation 3–6 h post‐treatment. RO6839921 and temozolomide, alone or in combination in mice implanted with *TP53* wt SHSY5Y‐Luc and NB1691‐Luc cells showed that combined RO6839921 and temozolomide led to greater tumour growth inhibition and increase in survival compared to vehicle control. Overall, RO6839921 had a favourable pharmacokinetic profile consistent with intermittent dosing and was well tolerated alone and in combination. These preclinical studies support the further development of idasanutlin in combination with temozolomide in neuroblastoma in early phase clinical trials.

## Abstract 2

**What's new?**

Long‐term survival of high‐risk neuroblastoma patients currently averages than 50%. New therapies that both improve survival and reduce treatment toxicity are urgently needed. MDM2 antagonists are a novel class of anti‐cancer agents that stabilize the p53 pathway and lead to tumour suppression. In this preclinical study, the authors tested a prodrug of the MDM2 inhibitor idasanutlin in mice. They found that this compound inhibited tumour growth and increased survival, especially in combination with temozolomide. These results support the further development of idasanutlin plus temozolomide in clinical trials for neuroblastoma.

**Abbreviations**
%ILS: percentage increase in lifespan
DMSO: Dimethyl sulfoxide
FDR: false discovery rate
GSEA: Gene Set Enrichment Analysis
H: hour(s)
IV: intravenous
LC–MS: liquid chromatography‐mass spectrometry
MGMT: O^6^‐methylguanine‐DNA methyltransferase
NOM: nominal *p*‐value
PBS: phosphate buffered saline
PD: pharmacodynamic
PK: pharmacokinetic
wt: wild‐type

## Introduction

Long‐term survival of high‐risk neuroblastoma patients (metastatic disease over 1 year of age or *MYCN* amplified disease) currently remains less than 50%, with those that survive often suffering long‐term side effects as a consequence of high‐dose intensive multimodal treatment.^1^ The identification of novel therapies that improve survival and reduce toxicity is urgently needed.

MDM2 antagonists are a novel class of anti‐cancer agents which, by disrupting the interaction between MDM2 and p53, lead to stabilisation and activation of the p53 pathway and wt p53 mediated tumour suppression through cell cycle arrest and apoptosis.^2^ Idasanutlin (RO5503781/RG7388), a pyrrolidine and second generation MDM2 antagonist from Hoffman‐La Roche has enhanced potency, selectivity and bioavailability, and has been developed in both oral and intravenous (IV; RO6839921) formulations.^3^, ^4^ To overcome tolerability issues with daily administration, intermittent schedules of idasanutlin, designed to enable bone marrow recovery, are being clinically evaluated in adult cancers both alone and in combination.^5^

*TP53* mutations are rare in neuroblastoma even at relapse, however upstream p53 pathway inactivation through *MDM2* amplification (2.5–7%) and *p14* ^*ARF*^ abnormalities (2–22% homozygous deletion; 7% methylation), have been reported particularly at relapse and support the use of MDM2 antagonists.^6^, ^7^, ^8^, ^9^ We and others have demonstrated highly potent anti‐tumour effects of idasanutlin in preclinical neuroblastoma models, alone and in combination with chemotherapy currently used in the treatment of high‐risk neuroblastoma, namely cisplatin, doxorubicin, topotecan (induction), busulfan (consolidation) and temozolomide (relapse),^10^, ^11^ and recently combinations with other targeted agents have been reported.^12^, ^13^ In addition, another MDM2 antagonist in clinical trials, MI‐773 (SAR405838), has been shown to enhance doxorubicin mediated cytotoxicity in neuroblastoma cell lines.^14^ To extend our original *in vitro* observations,^10^ our study assessed RO6839921 the IV prodrug of idasanutlin, alone and in combination with temozolomide, equivalent to one cycle of treatment, in 2 orthotopic models of neuroblastoma. RO6839921 (RG7775) is a pegylated IV prodrug of idasanutlin, which is rapidly metabolised by blood esterases to release active idasanutlin after administration, and was developed to reduce the variability in exposure and dose‐limiting gastrointestinal toxicity observed with oral idasanutlin, and for patients unable to tolerate capsules.^4^, ^15^, ^16^ Temozolomide is an alkylating agent which is part of standard backbone chemotherapy regimens for relapsed and refractory neuroblastoma.^17^ The current BEACON trial (ClinicalTrials.gov: NCT02308527) aims to test whether temozolomide and irinotecan is superior to temozolomide alone in the management of relapsed or refractory neuroblastoma. In our study we assessed the effect of temozolomide alone and in combination with RO6839921 (RG7775) in 2 orthotopic models of neuroblastoma prior to clinical evaluation.

## Materials and Methods

### Chemicals, cell lines and growth inhibition assays

Idasanutlin and the IV prodrug RO6839921 were kindly provided by Hoffman‐La Roche (Basel, Switzerland),^3^ and temozolomide was purchased from Sigma‐Aldrich (St. Louis, MO, USA). For *in vitro* studies, idasanutlin and temozolomide were dissolved in dimethyl sulfoxide (DMSO) (Sigma‐Aldrich). For *in vivo* studies, temozolomide was dissolved in PBS and clinically formulated RO6839921 reconstituted in water, immediately prior to use. Human *TP53* wt neuroblastoma cell lines SHSY5Y (non‐*MYCN* amplified) and NB1691 (*MYCN* and *MDM2* amplified) were retrovirally transduced with firefly luciferase as previously described,^18^ generating SHSY5Y‐Luc and NB1691‐Luc cell lines respectively. Cell lines were routinely confirmed to be Mycoplasma negative using MycoAlert™ (Lonza, Basel, Switzerland) and independently authenticated by multiplex short tandem repeat profiling by BMR Genomics (Padova, Italy) and NewGene Limited (Newcastle upon Tyne, UK) using Promega PowerPlex® Fusion System and GenePrint® 10 System, respectively. Seventy‐two hours growth inhibition assays and median effect analysis were performed as previously described.^10^

### Orthotopic *in vivo* experiments

All *in vivo* experiments were approved by the licencing and ethical committee of the IRCCS San Martino‐IST (Genoa, Italy), the Italian Ministry of Health, Newcastle University and the UK Government Home Office. Orthotopic murine SHSY5Y‐Luc or NB1691‐Luc models were established and monitored using bioluminescence (BLI) IVIS imaging, as previously described.^18^, ^19^, ^20^ RO6839921 (equivalent to 100 mg/kg of active idasanutlin) and temozolomide (34 mg/kg equivalent to 100 mg/m^2^ were given by IV tail vein injection and oral gavage, respectively. Tumour bearing mice were randomised into n = 3/group and n = 8/group for pharmacokinetic (PK)/pharmacodynamic (PD) studies and efficacy studies, respectively, and samples taken at the indicated time points (Supporting Information Fig. S1). To minimise esterase activity, 0.2% dichlorvos (Sigma‐Aldrich) was added to blood samples. All animals received the complete schedule of treatment without any signs of systemic toxicity. Animals were monitored 2–3 times weekly and euthanized humanely just before showing signs of illness/suffering such as abdominal dilatation, paraplegia, dehydration, or severe weight loss. Percentage increase in lifespan (%ILS) was calculated based on both median and mean survival to humane endpoint, as previously described.^5^ For more detailed procedures see Supporting Information.

### Western blotting, macrophage inhibitory cytokine‐1 (MIC‐1) ELISA assays and immunohistochemistry (IHC)

Tumours were disaggregated in Phosphosafe buffer (Merck Millipore) using the Medimachine with Medicons inserts (BD Biosciences, Oxford, UK) and Western analysis carried out as previously described.^21^ Human MIC‐1 assays were performed according to manufacturer's protocols (R&D Systems). IHC was performed using validated protocols and the Ventana Benchmark Ultra automated system (Ventana Medical Systems, Inc, Tucson, AZ, USA) in the Cellular Pathology Department, Royal Victoria Infirmary, Newcastle upon Tyne, UK.

### Pharmacokinetic analyses

Chromatographic separation of idasanutlin was achieved using a Prominence high‐performance liquid chromatography system (Shimadzu, Kyoto, Japan) with a Kinetex C18 50 mm x 4.6 mm 2.6 μm and a SecurityGuard cartridge C18 3 mm guard column (Phenomenex, California, USA) and an API4000 triple quadruple liquid chromatography‐mass spectrometry (LC–MS/MS) (Applied Biosystems, California, USA; Supporting Information; Table S1)**.** Aliquots of 20 μL plasma or tumour homogenate were used for analysis. PK data were analysed *via* non‐compartmental analysis using WinNonlin 6.3 (Pharsight, Princeton, NJ, USA).

### RNA‐Seq

NB1691 cells were treated for 24 h and 72 h with 1x their respective 72 h GI_50_ concentrations for idasanutlin (41 nM) and temozolomide (868.1 μM),^10^ both alone and in combination, or an equal volume of DMSO. Experiments were performed in triplicate. Total RNA was extracted using RNeasy Mini kit (Qiagen, Hilden, Germany) and RNA quantity and integrity assessed using the Agilent Bioanalyzer with RNA 6000 Nano Kit according to the manufacturer's instructions. RNA‐Seq was performed by Eurofins Genomics (Ebersberg, Germany). In brief, libraries were prepared using the Illumina TruSeq Stranded mRNA Library Prep Kit and sequenced at 2 x 100 bp using the Illumina HiSeq 2500 with v4 chemistry to achieve ∼30 M paired end reads per sample (NCBI GEO accession number: GSE104917). Reads were aligned against transcripts and gene level counts obtained using Salmon (https://combine-lab.github.io/salmon/) and R package tximport, respectively. Differential expression analysis was carried out using R package DESeq2. Gene Ontology analyses were conducted using The Database for Annotation, Visualisation and Integrated Discovery (DAVID) v6.8.^22^ Gene Set Enrichment Analysis (GSEA) using MSigDB h.all.v6.0.symbols.gmt [Hallmarks] was performed on minimally filtered preranked gene lists based on Log2 fold change relative to the relevant DMSO time point control.^23^ Gene sets with a false discovery rate (FDR) <25% and nominal (NOM) *p* value <0.05 were considered significant and enrichment plots visually inspected. Venn diagrams were generated using http://bioinformatics.psb.ugent.be/webtools/Venn/ to identify common gene sets between treatment conditions.

### Statistical analyses

All statistical tests were 2‐sided and performed using GraphPad Prism v6.0 software with *p* < 0.05 taken as the level of significance.

## Results

### Idasanutlin synergises with temozolomide in SHSY5Y‐Luc and NB1691‐Luc neuroblastoma cells *in vitro*

**Figure 1:**
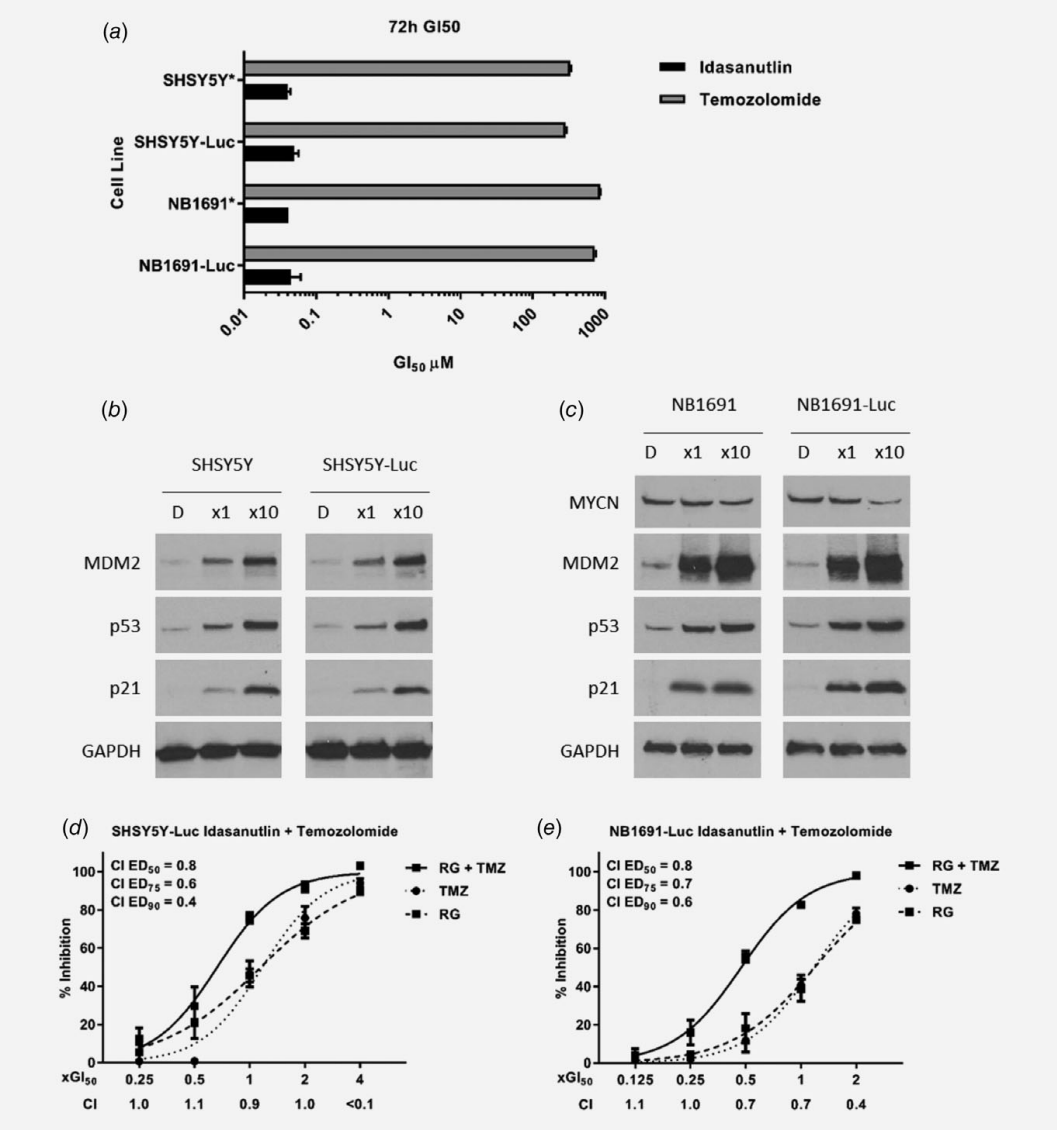
The effect of idasanutlin alone and in combination with temozolomide in parental and luciferase transduced neuroblastoma cell lines. (*a*) 72 h GI_50_ concentrations of parental and luciferase‐tagged cell lines to idasanutlin and temozolomide. Values represent the mean of n = 3 ± SEM. * as previously reported in Ref. 10. Western analysis for activation of the p53 pathway in (*b*) SHSY5Y and SHSY5Y‐Luc cells, and (*c*) NB1691 and NB1691‐Luc cells treated for 24 h with 1× and 10× their respective idasanutlin GI_50_ concentrations. D, DMSO treated control cells. Primary antibodies: p53 1:1000 (DO7, Leica Biosystems Ltd), MYCN 1:500 (sc‐53,993, Santa Cruz Biotechnology Inc., Dallas, TX, USA), MDM2 1:200 (OP46, Merck), p21^WAF1^ 1:200 (OP64, Merck), and GAPDH 1:500 (sc‐25,778, Santa Cruz Biotechnology Inc). Growth inhibition curves of (*d*) SHSY5Y‐Luc and (*e*) NB1691‐Luc exposed to idasanutlin and temozolomide alone and in combination at the indicated constant 1:1 ratios relative to their respective GI_50_ concentrations for 72 h. Data are shown as the average of at least 3 independent experiments and error bars represent SEM. CI values are shown for each constant ratio combination and also at ED_50_, ED_75_, and ED_90_. CI range: < 0.1 very strong synergism; 0.1–0.3 strong synergism; 0.3–0.7 synergism; 0.7–0.85 moderate synergism; 0.85–0.9 slight synergism; 0.9–1.1 nearly additive; 1.1–1.2 slight antagonism; 1.2–1.45 moderate antagonism; 1.45–3.3 antagonism; 3.3–10 strong antagonism; > 10 very strong antagonism. RG, idasanutlin; TMZ, temozolomide; RG + TMZ, idasanutlin and temozolomide.

To extend our previous *in vitro* observations of synergy between idasanutlin and temozolomide in a panel of 5 neuroblastoma cell lines^10^ into an orthotopic *in vivo* model of neuroblastoma, retrovirally transduced luciferase‐tagged SHSY5Y‐Luc and NB1691‐Luc, which can be readily detected using bioluminescence imaging, were generated. Both parental SHSY5Y and NB1691 cells have previously been shown to express high protein levels of O^6^‐methylguanine‐DNA methyltransferase (MGMT), which is associated with resistance to temozolomide.^10^, ^24^ Similar MGMT levels (Fig. S2, Supporting Information), sensitivities to idasanutlin and temozolomide (Fig. 1 *a*), and idasanutlin induced p53 pathway activation were confirmed in SHSY5Y‐Luc and NB1691‐Luc cells as parental cells (Fig. 1 *b*, *c*). Furthermore, median‐effect analysis was performed *in vitro* in SHSY5Y‐Luc (Fig. 1 *d*) and NB1691‐Luc cells (Fig. 1 *e*) and confirmed the previously reported synergistic interaction between idasanutlin and temozolomide in parental SHSY5Y and NB1691 cells, with comparable combination index (CI) values.^10^

### Pharmacokinetic profiling of RO6839921 in a *TP53* wt orthotopic model of neuroblastoma

Initial PK profile analysis of RO6839921 was conducted in SHSY5Y‐Luc orthotopic tumour bearing mice treated intravenously with a single dose of RO6839921 (equivalent to 100 mg/kg of active idasanutlin). Plasma samples were harvested at 15 min, 30 min, 1 h, 3 h, 6 h and 24 h post‐treatment, with tumours also harvested at 3, 6 and 24 h post‐treatment. In addition, analyses of plasma and tumour samples harvested 24 h after a single dose of temozolomide or RO6839921 and temozolomide in combination were performed, in order to assess any unexpected interactions between RO6839921 and temozolomide.

**Figure 2:**
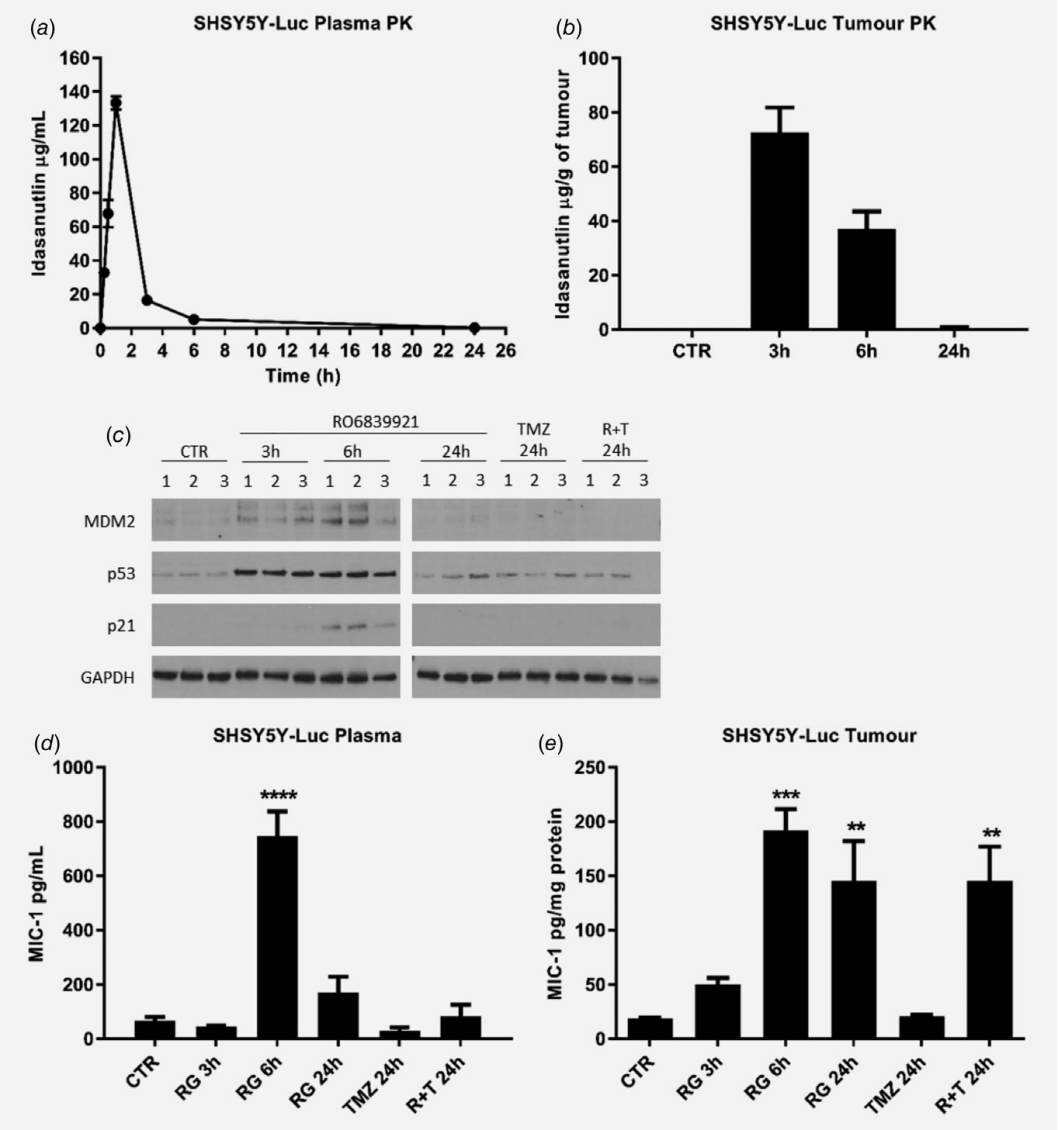
PK and PD analysis of RO6839921 in SHSY5Y‐Luc orthotopic tumour bearing mice. PK analysis of active idasanutlin levels using LC–MS in (*a*) plasma and (*b*) tumours harvested at the indicated time points from SHSY5Y‐Luc tumour bearing mice treated with a single dose of RO6839921 (equivalent to 100 mg/kg of active idasanutlin). PD profiling for (*c*) induction of the p53 pathway using Western analysis of p53, p21, and MDM2 levels in tumours, and MIC‐1 levels in (*d*) plasma and (*e*) tumours harvested at the indicated time points from SHSY5Y‐Luc tumour bearing mice after a single dose of RO6839921 (equivalent to 100 mg/kg of active idasanutlin), temozolomide (34 mg/kg) or RO6839921 and temozolomide. n = 3 mice per time point/group. CTR, control; RG, RO6839921; TMZ, temozolomide; R + T, RO6839921 and temozolomide. Primary antibodies: p53 1:1000 (#9282, Cell Signalling), MDM2 1:500 (sc‐813, Santa Cruz Biotechnology Inc), p21 1:1000 (#2947, Cell Signalling) and GAPDH 1:500 (sc‐25,778). Statistically significant differences were determined by one‐way analysis of variance (ANOVA) with Bonferroni post‐hoc tests and paired testing *vs*. control. *p* ≤ 0.05 (*); 0.01 (**); 0.001 (***); 0.0001 (****).

Detection of free idasanutlin showed increasing plasma idasanutlin levels over time, with peak levels observed 1 h post‐treatment (*C*_max_ = 133 ± 7 μg/mL), before returning almost to baseline by 24 h (0.1 ± 0.04 μg/mL), with a half‐life of 3.2 ± 0.5 h (Fig. 2 *a*; Table 1). The highest tumour idasanutlin levels were detected 3 h post‐treatment (18.2 ± 2.3 μg/mL), decreasing to approximately half by 6 h (9.3 ± 1.6 μg/mL) and returning almost to baseline by 24 h (0.16 ± 0.1 μg/mL) (Fig. 2 *b*). In line with plasma and tumour concentrations of RO6839921 alone at 24 h, similar minimal levels of idasanutlin were detected in plasma and tumour samples harvested 24 h post‐treatment with RO6839921 and temozolomide in combination. No idasanutlin was detected in plasma or tumour samples taken 24 h post‐treatment with temozolomide alone as expected.

**Table 1:** Idasanutlin pharmacokinetic parameters after administration of a single dose of RO6839921 (equivalent to 100 mg/kg)

Mouse	C_max_ (mg/L)	AUC_last_ (h*mg/L)	AUC_inf_ (h*mg/L)	T_1/2_ (h)
1	141	326	327	2.9
2	128	295	295	3.0
3	141	280	281	3.7
Mean	133	300	301	3.2
Standard Deviation	7	24	23	0.5

A minimum of three data points were used to calculate λz with uniform weighting. Area under the curve (AUC) was calculated using the linear trapezoidal method.

### RO6839921 induces activation of the p53 pathway in a *TP53* wt orthotopic model of neuroblastoma

Inhibition of the interaction between MDM2 and p53 leads to stabilisation of p53 and subsequent p53 mediated transcriptional upregulation of downstream target genes such as *p21*, *MDM2* and *MIC‐1*. PD profiling of RO6839921 was therefore conducted on plasma and tumour samples harvested at 3, 6 and 24 h post‐treatment using Western analysis for p53, p21 and MDM2, and MIC‐1 ELISA assays (Fig. 2 *c*–*e*). Tumour analysis showed maximal p53 stabilisation from 3 h, sustained until 6 h post‐treatment, with peak induction of both MDM2 and p21 at 6 h, and all levels returning almost to baseline by 24 h (Fig. 2 *c*). Consistent with the latter, a marginal increase in p53 above baseline with no p21 or MDM2 induction was observed in samples harvested 24 h post‐treatment with temozolomide alone and the combination (Fig. 2 *c*).

MIC‐1 is a secreted protein generated from a transcriptional target gene of p53, and thus readily detectable in blood. Elevated serum MIC‐1 levels have previously been shown to be a potential surrogate PD biomarker of MDM2 inhibitor activity in RG7112 and idasanutlin clinical trials.^25^, ^26^, ^27^ Plasma and tumour samples were analysed using a human MIC‐1 ELISA assay, therefore any detected MIC‐1 is tumour specific (Fig. 2 *d*, *e*). In plasma, MIC‐1 levels were unchanged at 3 h, peaked at 6 h and returned almost to baseline by 24 h post‐treatment. No elevation of MIC‐1 levels was detected in plasma samples harvested 24 h post‐treatment with temozolomide alone, and MIC‐1 levels in samples harvested 24 h post‐treatment with RO6839921 and temozolomide were slightly lower than those observed in 24 h RO6839921 alone samples (Fig. 2 *d*). In tumour samples, elevated MIC‐1 levels were detected from 3 h post‐treatment, peaked at 6 h and decreased slightly by 24 h. No elevated MIC‐1 levels were detected in tumour samples harvested 24 h post‐treatment with temozolomide alone. MIC‐1 levels in tumour samples harvested 24 h post‐treatment with RO6839921 and temozolomide were comparable to the 24 h RO6839921 alone samples (Fig. 2 *e*). Peak MIC‐1 plasma and tumour levels observed at 6 h post‐treatment are consistent with the peak MDM2 and p21 induction observed by protein analysis (Fig. 2 *c*).

### RO6839921 in combination with temozolomide induces tumour growth inhibition and an increase in lifespan

The efficacy of RO6839921 alone and in combination with temozolomide was assessed in SHSY5Y‐Luc and NB1691‐Luc orthotopic tumour bearing mice. The intermittent schedule used is equivalent to one cycle of treatment over a 3 week period (Fig. S1, Supporting Information). Mice were randomised into control, RO6839921 (once per week for 3 weeks), temozolomide (daily x5/3 weeks; consistent with the currently used clinical dosing schedule in the BEACON Trial (NCT02308527)), or RO6839921 and temozolomide treatment groups (n = 8/group; Fig. S1, Supporting Information). No weight loss was observed for the duration of treatment suggesting all treatments were well tolerated (Fig. S3A, B, Supporting Information).

**Figure 3:**
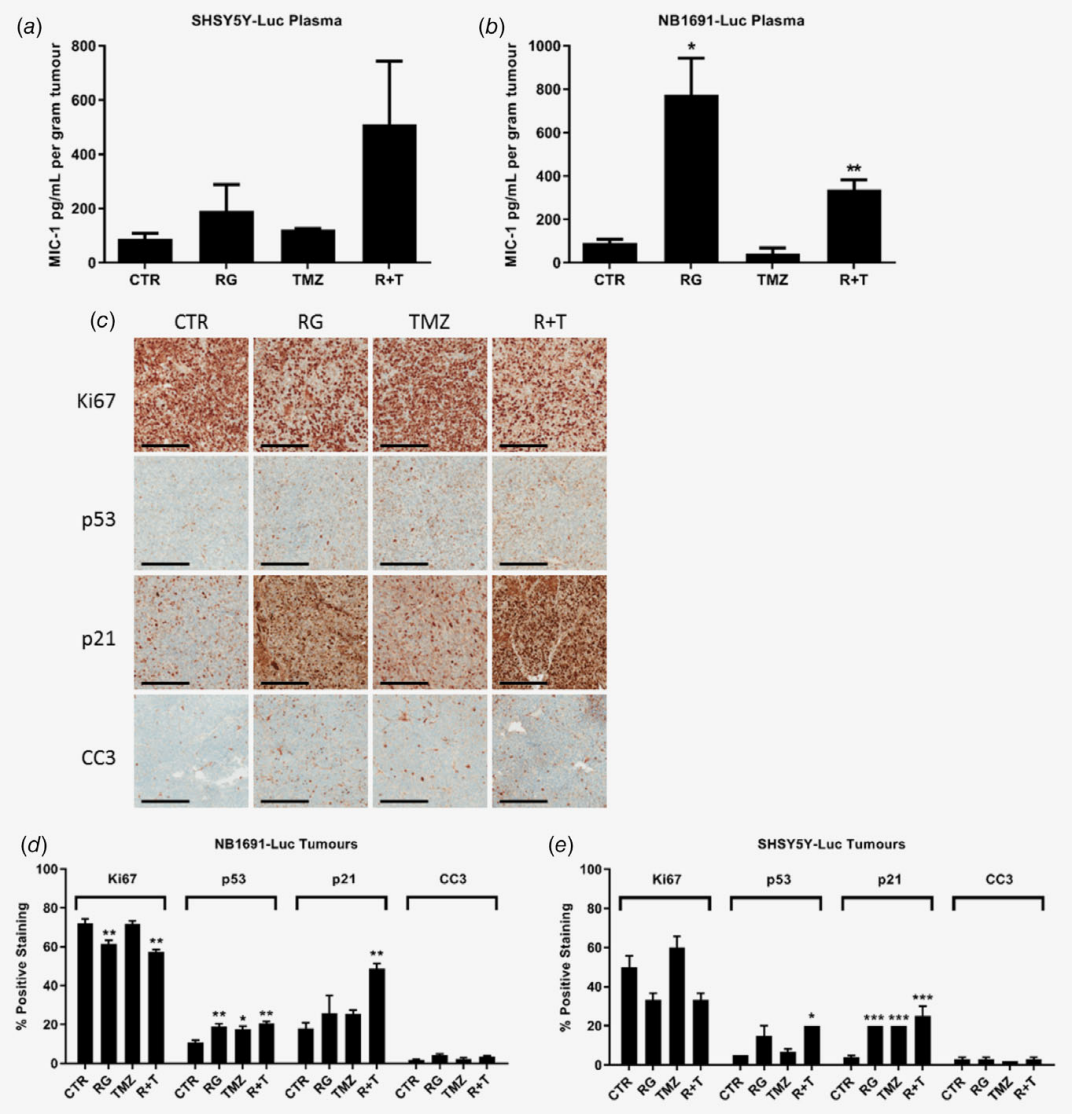
MIC‐1 levels and IHC analysis of Ki67, p53, p21, and cleaved caspase 3 (CC3) in orthotopic tumours in response to one cycle of RO6839921 and temozolomide alone and in combination. Plasma MIC‐1 levels in untreated and treated (*a*) SHSY5Y‐Luc and (*b*) NB1691‐Luc orthotopic tumour bearing mice taken 24 h after the last day of treatment. MIC‐1 levels were normalised to tumour weight, to account for variations in tumour size in response to treatment. Statistically significant differences *vs*. control were determined by unpaired *t*‐tests. (*c*) Representative images captured using the Aperio FL Digital Pathology Slide Scanner of Ki67 (Dako, Agilent, Stockport, UK), p53 (Bp53‐11, Ventana Medical Systems, Inc), p21 (OP64; Merck Millipore, Watford, UK), and CC3 (MAB835; R&D Systems, Abingdon, UK) stained 5 μm thick formalin‐fixed, paraffin embedded NB1691‐Luc tumour sections. Scale bar = 200 μm. Graphical representation of quantification of % positive Ki67, p53, p21, and CC3 staining in (*d*) NB1691‐Luc and (*e*) SHSY5Y‐Luc orthotopic tumours using Imagescope software and algorithms (Leica Biosystems, Newcastle upon Tyne, UK) and/or scored for positive immunostaining by a consultant pathologist (KW). n = 3 mice per group. All data are shown as the mean and error bars represent SEM. CTR, control; RG, RO6839921; TMZ, temozolomide; R + T, RO6839921 and temozolomide. Statistically significant differences were determined by one‐way ANOVA with Bonferroni post‐hoc tests and paired testing *vs*. control. *p* ≤ 0.05 (*); 0.01 (**); 0.001 (***); 0.0001 (****). [Color figure can be viewed at wileyonlinelibrary.com]

Twenty‐four hours after completion of one treatment cycle, 3 mice per group were sacrificed and plasma and tumours harvested for PK and PD analyses (Fig. 3; Fig. S1, Supporting Information). Plasma idasanutlin levels were comparable to initial PK data (Fig. 2 *a*; Fig. S3C, Supporting Information). In SHSY5Y‐Luc tumour bearing mice, although the combination treatment led to the greatest increase in plasma MIC‐1 levels, the variation between the triplicates was large possibly as a consequence of the small sample size (Fig. 3 *a*). Interestingly, in contrast NB1691‐Luc tumour bearing mice, RO6839921 alone led to the greatest and most statistically significant increase in plasma MIC‐1 levels (Fig. 3 *b*).

In addition, immunohistochemical analysis of harvested SHSY5Y‐Luc and NB1691‐Luc orthotopic tumours was performed to determine histology and assess treatment induced effects on the p53 pathway, proliferation and apoptosis (Fig. 3 *c*–*e*). Both SHSY5Y‐Luc and NB1691‐Luc orthotopic tumours were histologically comparable with poorly differentiated human neuroblastoma (Fig. S3D, E, Supporting Information). In NB1691‐Luc tumours, both RO6839921 alone and in combination with temozolomide led to a statistically significant reduction in Ki67 positive cells *vs*. control (Fig. 3 *c*, *d*). In contrast, temozolomide treatment alone did not reduce Ki67 immunostaining (Fig. 3 *c*, *d*). RO6839921 and temozolomide alone and in combination all resulted in significant increases in p53 positive cells. RO6839921 and temozolomide alone induced increases in p21 positivity, with combination treatment resulting in the greatest and statistically significant increase (Fig. 3 *c*, *d*). In SHSY5Y‐Luc tumours although RO6839921 alone and in combination with temozolomide led to a decrease in Ki67 staining this was not statistically significant (Fig. 3 *e*). There was an increase in p53 positivity in response to RO6839921 alone and in combination with temozolomide, but only the latter was statistically significant (Fig. 3 *e*). In contrast, a statistically significant increase in p21 positivity was observed in response to all treated groups (Fig. 3*e*). The overall positivity for cleaved caspase‐3 across all treatment groups in both models was low (≤5%) and remained relatively unchanged despite increases in p53 staining (Fig. 3 *c*–*e*). This is in contrast to our previous *in vitro* observations of higher caspase 3/7 activity in response to single agent and combination treatment. Additional γ‐H2AX (p‐serine 139) was performed as an indicator of DNA fragmentation and apoptosis.^28^ The results showed that all treatments resulted in increased γ‐H2AX IHC staining *vs*. control, however only RO6839921 alone led to a statistically significant increase in NB1691‐Luc tumours and temozolomide alone led to a statistically significant increase in SHSY5Y‐Luc tumours (Fig. S4A,B, Supporting Information). In contrast, a greater induction of γ‐H2AX (p‐serine 139) was observed in SHSY5Y cells treated *in vitro* with idasanutlin and temozolomide in combination than either agent alone (Fig. S4C, Supporting Information). Taken together the differences between *in vitro* and *in vivo* observations suggest it is possible that the timepoint at which tumours were harvested was suboptimal for detection of apoptosis by IHC and earlier timepoints may have been more informative.

**Figure 4:**
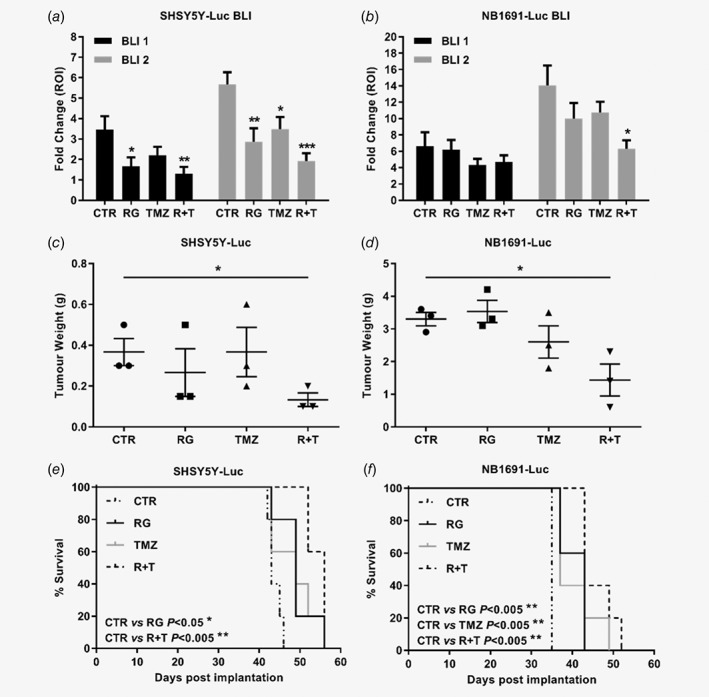
Efficacy of one cycle of RO6839921 alone and in combination with temozolomide in SHSY5Y‐Luc and NB1691‐Luc orthotopic tumour bearing mice. Graphical representation of bioluminescence emission of untreated (CTR) and treated (*a*) SHSY5Y‐Luc and (*b*) NB1691‐Luc orthotopic tumour bearing mice on Day 8 (BLI 1) and Day 15 (BLI 2) of treatment with RO6839921 alone (RG), temozolomide alone (TMZ) or RO6839921 and temozolomide in combination (R + T). n = 8 mice per group. Statistically significant differences *vs*. control were determined by unpaired *t*‐tests. Weight of tumours from untreated and treated (*c*) SHSY5Y‐Luc and (*d*) NB1691‐Luc orthotopic tumour bearing mice taken 24 h after the last day of treatment. n = 3 mice per group. All data points are shown as the mean and error bars represent SEM. Statistically significant differences *vs*. control were determined by unpaired *t*‐tests. Kaplan‐Meier plots and log‐rank (Mantel‐Cox) analysis of survival of control and treated (*e*) SHSY5Y‐Luc and (*f*) NB1691‐Luc orthotopic tumour bearing mice. n = 5 mice per group. CTR, control; RG, RO6839921; TMZ, temozolomide; R + T, RO6839921 and temozolomide. *p* ≤ 0.05 (*); 0.01 (**); 0.001 (***).

Efficacy of RO6839921 and temozolomide alone and in combination on tumour growth were monitored by bioluminescence imaging, tumour weight, survival and treatment induced %ILS (Fig. 4; Table 2; Fig. S1, Supporting Information). After 1 week of treatment (Day 8/BLI1), a 52% reduction in bioluminescence *vs*. control was observed in SHSY5Y‐Luc tumour bearing mice treated with RO6839921, 36% for temozolomide alone and 63% for the combination, where the latter led to the greatest and most statistically significant reduction (Fig. 4 *a*). A similar reduction in bioluminescence was observed after 2 weeks of treatment (Day 15/BLI2); 49% for RO6839921 alone, 39% for temozolomide alone and 66% for the combination, with all treatments producing statistically significant differences and the combination treatment again leading to the greatest and most significant reduction (Fig. 4 *a*; Fig. S5, Supporting Information). In NB1691‐Luc tumour bearing mice, after 1 week of treatment (Day 8/BLI1), a 9% reduction in bioluminescence *vs*. control was observed for RO6839921 alone, 35% for temozolomide alone and 29% for the combination, although none were statistically significant (Fig. 4 *b*). In contrast, after 2 weeks of treatment (Day 15/BLI2), a 29% reduction in bioluminescence was observed for RO6839921 alone, 24% for temozolomide alone and 55% for the combination, where the latter led to the greatest and a statistically significant reduction compared to control (Fig. 4 *b*; Fig. S5, Supporting Information). In addition, after 2 weeks of treatment (Day 15/BLI2), the combination also led to statistically significant reductions (unpaired *t*‐test, *p* < 0.05) in bioluminescence in both SHSY5Y‐Luc and NB1691‐Luc models *vs*. temozolomide alone.

**Table 2:** Percentage increase in lifespan (ILS) of SHSY5Y‐Luc and NB1691‐Luc orthotopic tumour bearing mice in response to treatment with RO6839921 and temozolomide alone and in combination

Cell line	Median/Mean/%ILS	Control	RO6839921	Temozolomide	RO6839921 + temozolomide
SHSY5Y‐Luc	Median Survival (Day)	43	49	49	56
	%ILS (Median)	‐	14%	14%	30%
	Mean Survival (Day)	44	49	49	54
	%ILS (Mean)	‐	12%	11%	24%
NB1691‐Luc	Median Survival (Day)	35	43	37	43
	%ILS (Median)	‐	23%	6%	23%
	Mean Survival (Day)	35	41	41	46
	%ILS (Mean)	‐	16%	16%	31%

In line with bioluminescence results, assessment of tumour weight harvested 24 h after the last treatment demonstrated the combination treatment led to the greatest and a statistically significant reduction in tumour weight in both SHSY5Y‐Luc (64% reduction; Fig. 4 *c*) and NB1691‐Luc (57% reduction; Fig. 4 *d*) orthotopic models compared to control. However, tumour weight reduction after combination treatment *vs*. temozolomide alone did not reach statistical significance in either model. *MYCN* amplified NB1691‐Luc cells grew more rapidly and formed larger orthotopic tumours compared to non‐*MYCN* amplified SHSY5Y‐Luc cells consistent with *MYCN* amplified disease (Fig. 4 *c*,*d*). No macroscopic metastases were observed in either model.

Finally, SHSY5Y‐Luc and NB1691‐Luc tumour bearing mice were monitored for survival to humane endpoint, and treatment induced increases in life span (% ILS) *vs*. control determined in the remaining n = 5/group (Fig. 4 *e*, *f*; Table 2, Fig. S1, Supporting Information). In the SHSY5Y‐Luc model, both RO6839921 alone (*p* < 0.05) and in combination (*p* < 0.005) led to significant increases in survival *vs*. control (Fig. 4 *e*), whereas in the NB1691‐Luc model, RO6839921 and temozolomide alone and in combination all led to significant increases in survival (*p* < 0.005; Fig. 4 *f*). Percentage ILS based on median survival *vs*. control was 14% for both RO6839921 and temozolomide alone, and 23% for the combination in the SHSY5Y‐Luc orthotopic model (Table 2). In the NB1691‐Luc orthotopic model, %ILS *vs*. control was 6% for temozolomide alone and 23% for both RO6839921 alone, and the combination. Although in the NB1691‐Luc model there was no difference in median survival between RO6839921 alone and the combination, NB1691‐Luc tumour bearing mice treated with the combination survived the longest (Fig. 4 *f*), and comparison of %ILS based on mean survival was 16% for RO6839921 alone *vs*. 31% for combination treatment (Table 2).

### Idasanutlin and temozolomide act through activation of the p53 pathway and inhibition of MYC targets

**Figure 5:**
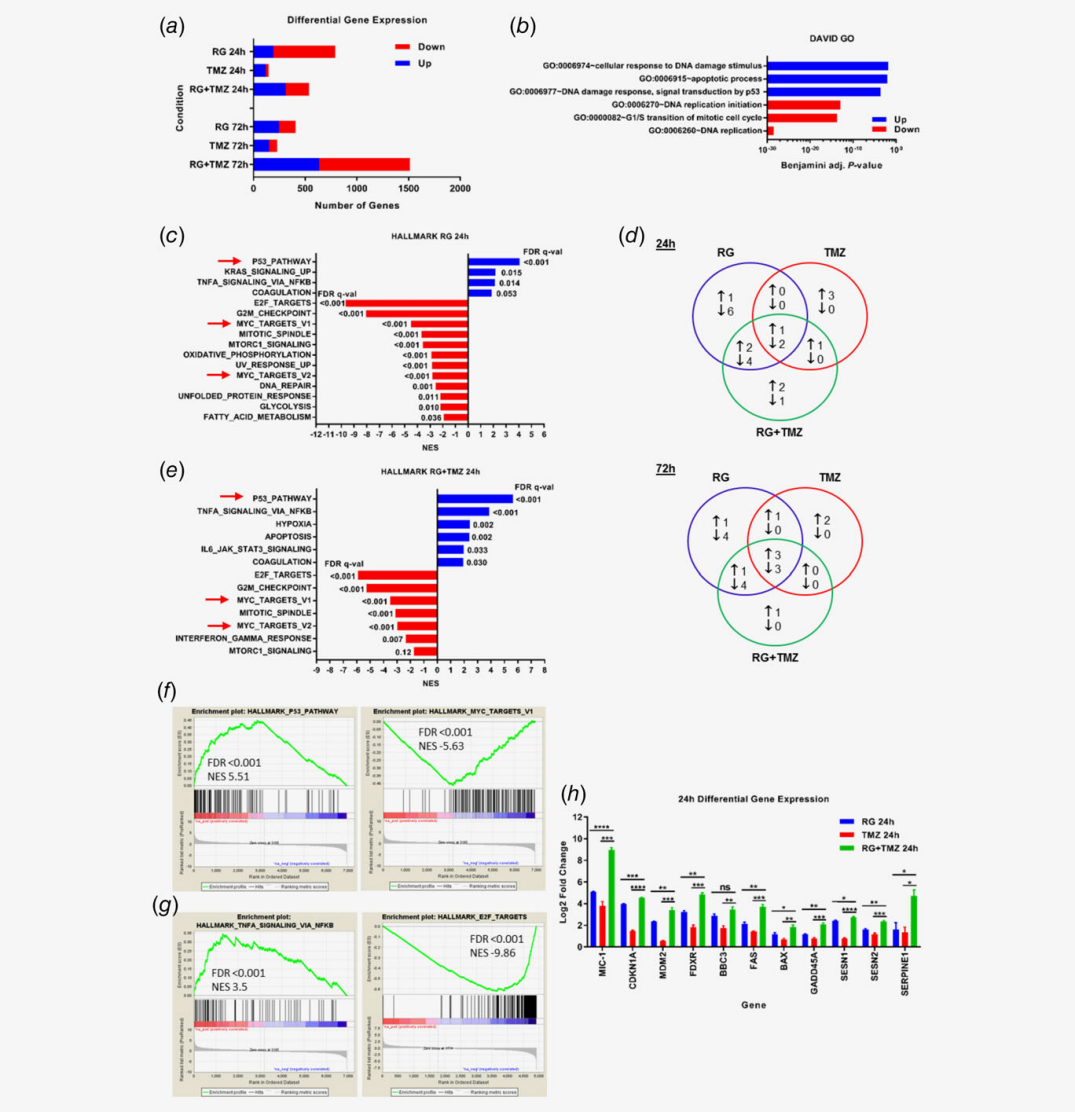
RNA‐Seq analysis of NB1691 cells treated with idasanutlin and temozolomide alone and in combination. (*a*) Graph showing the number of statistically significant and greater than two‐fold differentially expressed genes in response to idasanutlin and temozolomide alone and in combination for 24 h and 72 h. (*b*) Top 3 DAVID GO biological processes up‐ and downregulated in response to idasanutlin and temozolomide in combination for 24 h. (*c*) Enriched Hallmark gene sets identified using GSEA for genes up‐ and downregulated in NB1691 cells in response to idasanutlin for 24 h *vs*. DMSO. NES, Normalised Enrichment Score. Red arrows indicate gene sets common to all treatment conditions at 24 h. (*d*) Venn diagrams showing the overlap of gene sets enriched in the different treatment conditions at 24 h (top) and 72 h (bottom). (*e*) Enriched Hallmark gene sets identified using GSEA for genes up‐ and downregulated in NB1691 cells in response to idasanutlin and temozolomide in combination for 24 h. Red arrows indicate gene sets common to all treatment conditions at 24 h. GSEA gene set enrichment plots showing (*f*) positive enrichment of the p53 Pathway and negative enrichment of MYC Targets v1 and (*g*) positive enrichment of TNFA Signalling *via* NFKB and negative enrichment of E2F Targets in NB1691 cells treated with idasanutlin and temozolomide in combination for 72 h. (*h*) Graph showing the Log2 fold change in expression relative to DMSO control of selected p53 regulated genes of interest in NB1691 cells in response to idasanutlin and temozolomide alone and in combination for 24 h. Statistical significance was determined using unpaired *t*‐tests *p* ≤ 0.05 (*); 0.01 (**); 0.001 (***); 0.0001 (****); ns, not significant; RG, idasanutlin; TMZ, temozolomide; RG + TMZ, idasanutlin and temozolomide. [Color figure can be viewed at wileyonlinelibrary.com]

To identify potential pathways and mechanisms involved in idasanutlin and temozolomide combination treatment, RNA‐Seq was performed on NB1691 cells treated with idasanutlin and temozolomide alone and in combination for 24 h and 72 h. Differential gene expression analysis showed that idasanutlin alone resulted in the greatest number of differentially expressed genes *vs*. DMSO control at 24 h post‐treatment, while the combination resulted in the greatest number at 72 h post‐treatment (Fig. 5 *a*). Temozolomide alone led to the smallest number of differentially expressed genes *vs*. DMSO at both time points (Fig. 5 *a*). Consistent with this, analyses to determine the similarity between treatment samples showed clustering of idasanutlin with the combination, and DMSO control with temozolomide (Fig. S6, Supporting Information). Gene ontology analysis demonstrated that the combination led to upregulation of genes involved in p53, apoptosis and signal transduction and downregulation of genes involved in DNA replication, mitosis, cell cycle progression and cell division (Fig. 5 *b* and Fig. S7A, Supporting Information).

GSEA of idasanutlin treated NB1691 cells similarly confirmed upregulation of genes involved in the p53 pathway and also revealed downregulation of MYC targets and genes involved in metabolism and MTORC1 signalling (Fig. 5 *c* and Fig. S7B, Supporting Information). Comparison of overlapping gene sets between single and combination treatments at 24 h and 72 h post‐treatment are shown in Figure 5 *d*. This revealed that both idasanutlin and temozolomide led to upregulation of the p53 pathway and downregulation of MYC target genes at 24 h and 72 h (Fig. 5 *c*–f and Fig. S7B‐D, Supporting Information), with additional upregulation of genes involved in apoptosis and TNFA signalling *via* NFKB, and downregulation of E2F targets at 72 h (Fig. 5 *d*,*g* and Fig. S7B‐D, Supporting Information). These gene sets and pathways are consistent with the expected pro‐apoptotic and anti‐proliferative mechanism of action for combining an MDM2 inhibitor with a cytotoxic agent. The downregulation of MYC targets is particularly interesting as NB1691 cells are *MYCN* amplified, but interestingly MYCN protein levels remained unchanged after 24 h treatment with idasanutlin alone (Fig. 1 *c*).

Consistent with cluster analysis (Fig. S6B, Supporting Information), idasanutlin and the combination treatment also share additional gene sets not observed in response to temozolomide alone, in particular downregulation of genes involved in MTORC1 signalling was observed at 24 h and 72 h (Fig. 5 *c*–*e* and Fig. S7B‐D, Supporting Information). Analysis of gene sets enriched only in the combination treatment revealed upregulation of IL6‐JAK‐STAT3 Signalling and downregulation of genes regulated by Interferon Gamma at 24 h, suggesting a potential cellular stress response and immune cell modulation (Fig. 5 *d*,*e*).

In view of the identified cooperative upregulation of the p53 pathway by idasanutlin and temozolomide by GSEA, assessment of the differential expression of selected p53 regulated genes of interest showed that overall the combination led to significantly greater induction than either agent alone at 24 h and 72 h (Fig. 5 *h* and Fig. S7E, Supporting Information). This includes *MIC‐1*, *p21* and *MDM2* which are used as PD biomarkers in studies of MDM2 antagonists, including our study, as well as pro‐apoptotic genes *FDXR, BBC3, FAS, BAX* and *GADD45A*. Similarly*, SESN1* and *SESN2* increased, both negative regulators of MTORC1 consistent with the observed downregulation of MTORC1 Signalling,^29^ and *SERPINE1,* which is involved in, and acts as a marker for replicative senescence^30^ (Fig. 5 *h* and Fig. S7E, Supporting Information).

## Discussion

Novel therapies to improve survival of high‐risk neuroblastoma patients are urgently needed. Our study is the first to evaluate and demonstrate the efficacy of RO6839921, the IV prodrug of the MDM2 antagonist idasanutlin, as a single agent and in combination with temozolomide in 2 *TP53* wt orthotopic models of neuroblastoma. This follows on from our previous extensive *in vitro* assessment of idasanutlin alone and in combination with chemotherapies (cisplatin, doxorubicin, topotecan, busulfan and temozolomide) in a panel of *MYCN* amplified and non‐amplified neuroblastoma cell lines.^10^ The intermittent dosing schedule tested showed that both RO6839921 alone and in combination with temozolomide were well tolerated. However, some MDM2 antagonists have been reported to display interspecies selectivity, with reduced binding affinities for mouse and rat MDM2,^31^ thus further investigation of RO6839921 based combinations in suitable toxicity models may be required.

*In vivo* efficacy data, incorporating a combination of tumour bioluminescence, tumour weight and survival analyses in both models, together demonstrated that a single cycle of RO6839921 was as efficacious as temozolomide alone, and when used in combination led to the greatest tumour growth inhibition, as evident by bioluminescence (Day15/BLI2) and tumour weight reduction, and increase in survival. In comparison to temozolomide alone, the combination resulted in statistically significant reductions in bioluminescence (Day 15/BLI2) in both models. Minor differences in response to treatments between the 2 orthotopic models used could potentially be attributed to *MYCN* amplification in NB1691 cells which led to *in vivo* tumours that formed more rapidly and were larger in size. Taken together, the observed *in vivo* efficacy is in keeping with our previous *in vitro* synergy data of idasanutlin and temozolomide, with the combination demonstrating clear evidence of activity after only one cycle of intermittent treatment. For clinical evaluation, multiple cycles and testing or other dosing schedules are recommended such as the adult RO6839921 schedule (daily ×5/ 28 day cycles; NCT02098967).

Our study also provides valuable PK and PD data to support paediatric clinical trials with an observed PK profile of RO6839921 consistent with IV administration, and compatible with recommended intermittent dosing schedules aimed to enable bone marrow recovery and overcome haematological toxicities. The PD profile was consistent with the PK profile, mechanism of action, and previous studies.^11^, ^25^, ^27^, ^32^ The analysis of PD biomarkers of response to RO6839921 alone showed changes in MIC‐1, p21, p53 and Ki67, consistent with our previous *in vitro* observations of idasanutlin mediated anti‐tumour activity.^10^ Our study is the only preclinical study of idasanutlin in neuroblastoma so far to include MIC‐1 as a PD biomarker, and demonstrated that in response to combination treatment MIC‐1 was the most responsive biomarker in the SHSY5Y‐Luc model and p21 in the NB1691‐Luc model. RNA‐Seq analysis of NB1691 cells *in vitro* showed the greatest induction of *MIC‐1* expression in response to combination treatment compared to either idasanutlin or temozolomide alone. Since circulating MIC‐1 is not tumour specific, we recommend that future studies continue to include a panel of PD biomarkers for proof‐of‐mechanism and response, ideally including p21 tumoural or circulating tumour cell expression as recently reported by us (Merugu *et al*, *submitted*).

We previously conducted similar PK/PD analyses of orally formulated idasanutlin in preliminary studies of a subcutaneous NGP neuroblastoma xenograft model after a single oral dose equivalent to 100 mg/kg of active idasanutlin (Fig. S8, Supporting Information). Comparison of the present RO6839921 PK and PD data with orally formulated idasanutlin demonstrates that consistent with the route of administration and previous literature,^4^ RO6839921 led to a much higher plasma concentration of active idasanutlin (>100 μg/mL *vs*. ∼5 μg/mL). Moreover, PD analysis showed a more consistent and robust induction of PD biomarkers in response to RO6839921 compared to oral idasanutlin, at least partly as a result of the higher achievable plasma concentrations. These data therefore provide support for the superior PK profile and activity of RO6839921.

High MGMT levels are usually the primary cause of temozolomide resistance, with deficient MMR also playing a role.^33^ The role of p53 in temozolomide sensitivity has not been fully elucidated, however p53 is known to be involved in MMR and the DNA damage response and has previously been shown to induce downregulation of *MGMT* through sequestration of the Sp1 transcription factor.^34^, ^35^ Although no decrease in *MGMT* mRNA levels in response to idasanutlin was observed in our study, a decrease in MGMT protein levels was observed and suggests post‐translational mechanisms (Fig. S9, Supporting Information). GSEA revealed that idasanutlin and temozolomide act cooperatively to upregulate the p53 pathway and downregulate MYC targets, the former resulting in significantly greater induction of several apoptotic genes. Furthermore, the data also revealed idasanutlin mediated downregulation of MTORC1 signalling which has previously been linked to chemoresistance and could therefore sensitise cells to temozolomide. However, a recent Phase II trial of irinotecan‐temozolomide with temsirolimus in children with refractory/relapsed neuroblastoma did not observe outcomes meriting further study.^36^ A recent study has reported potent preclinical efficacy combining the MTORC1 inhibitor temsirolimus with idasanutlin in neuroblastoma preclinical models.^12^ Temozolomide has previously been reported to be a substrate of MDR‐1, which is expressed at high levels in both SHSY5Y and NB1691 cells and we have previously reported that at very high concentrations idasanutlin is able to modulate MDR‐1 function.^21^, ^37^ Taken together, these provide potential mechanisms by which MDM2 antagonists enhance temozolomide mediated cytotoxicity, consistent with the observed efficacy of RO6839921 combined with temozolomide.

An increasing number of targeted agents are now entering early phase paediatric trials including the lead Novartis MDM2 antagonist, HDM‐201, as part of the Next Generation Personalised Neuroblastoma Therapy (NEPENTHE) trial (NCT02780128) and the dual MDM2/MDMX inhibitor ALRN‐6924 (NCT03654716). Additionally, Alisertib (Aurora kinase A) and Crizotinib (ALK) have progressed to Phase I/II trials in combination with chemotherapy in neuroblastoma patients^38^, ^39^, ^40^ and the benefit of incorporating Crizotinib to upfront standard therapy for patients with newly diagnosed high‐risk neuroblastoma will be evaluated in an upcoming Phase III trial (NCT03126916). Full results from the recently completed Phase I trial of RO6839921 in adults with advanced solid tumours and AML are pending (NCT02098967). However, interim data from the advanced solid tumour arm of the trial show that RO6839921 was generally well tolerated and main dose limiting toxicities observed were thrombocytopenia and neutropenia, in line with adult trial data for oral idasanutlin, as well as other MDM2 inhibitors.^16^, ^41^, ^42^, ^43^ Gastrointestinal toxicity was reported but not dose limiting as previously reported with oral Idasanutlin.^15^, ^16^ The current emphasis is on the development of the oral formulation of idasanutlin, which in comparison to the IV formulation, is at a later stage of development (Phase 3 in AML). Temozolomide is currently used as standard backbone chemotherapy regimens for relapsed and refractory neuroblastoma and has been shown to be generally well tolerated. Clinical data in children have reported thrombocytopenia and neutropenia as the main toxicities.^44^, ^45^, ^46^, ^47^ Taken together with the above reported toxicities for RO6839921 and idasanutlin,^15^, ^16^ potential toxicities for RO6839921/idasanutin with temozolomide are anticipated to be haematological and gastrointestinal, but manageable, particularly if temozolomide is used at the lower combination regimen dose of 100 mg/m^2^.

Based on our previous *in vitro* assessment of idasanutlin^10^ and in line with the inclusion criteria for current clinical trials of MDM2 inhibitors, we recommend that selection of patients be based on the presence of wt *TP53*, however patient *p14* ^*ARF*^ and *MDM2* status should be recorded. Baseline tumour MDM2 expression may be important as clinical response to idasanutlin in AML patients has been associated with baseline MDM2 expression in leukaemic blasts.^48^ In conclusion, our study provides valuable PK, PD and efficacy data of IV RO6839921 as well as potential mechanisms of synergy to support the paediatric assessment of IV or oral idasanutlin, alone and in combination with a temozolomide backbone to provide a novel therapeutic strategy for patients with relapsed/refractory high‐risk neuroblastoma.

## Conflict of interest

We disclose that L. Chen and D.A. Tweddle are part of an international collaborative research consortium with Hoffmann‐La Roche Ltd. Newcastle University, Cancer Research Technology and Astex Pharmaceuticals Inc. are part of an alliance agreement since 2012 and D.R. Newell, D. A Tweddle and J. Lunec have received research support and funding from Astex Pharmaceuticals, Inc. All other authors declare no conflicts of interest.

## Supporting information


**Appendix S1:** Supporting Information
