# Supplementary material for: Preclinical evaluation of the first intravenous small molecule MDM2 antagonist alone and in combination with temozolomide in neuroblastoma
Source: Int J Cancer. 2019 Jan 9;144(12):3146–59. doi: 10.1002/ijc.32058 (PMC6491995; doi:10.1002/ijc.32058)
Supplement: Supplementary file 1 — Appendix S1: Supporting Information [file IJC-144-3146-s001.pdf]

# Supplementary Information

## *Detailed methods of orthotopic in vivo experiments*

Five week old female athymic Nude-Foxn1$^{nu}$ mice (Envigo, Bresso, Italy) were anesthetized, subjected to a laparotomy and orthotopically injected with 1×10$^6$ SHSY5Y-Luc or NB1691-Luc neuroblastoma cells into the capsule of the left adrenal gland, as previously described (1-3). The lethality of the method was 0%, and mice were monitored using BLI IVIS imaging for tumour development (~2-3 weeks) prior to commencement of treatment. For PK and PD studies, mice implanted with SHSY5Y-Luc cells were treated with a single dose of RO6839921 (equivalent to 100mg/kg of active idasanutlin) via tail vein injection, temozolomide (34mg/kg) via oral gavage or RO6839921 and temozolomide in combination (n=3 mice/group). For RO6839921 alone, blood samples were taken at 15, 30 and 60min post-treatment via tail vein bleeds, with terminal blood and tumour samples collected at 3, 6 and 24h post-treatment. For temozolomide alone and the combination treatment, plasma and tumours were harvested only at 24h post-treatment. Tumours were snap frozen and stored in liquid nitrogen for subsequent analyses. To minimize esterase activity, 0.2% dichlorvos (Sigma-Aldrich) was added to blood samples, plasma samples harvested by centrifugation at 1500 x g for 10min and stored at -80°C. For efficacy studies, SHSY5Y-Luc or NB1691-Luc tumour bearing mice were randomized into control, RO6839921 (equivalent to 100mg/kg of active idasanutlin, once per week x3 by IV tail vein injection), temozolomide (34mg/kg, daily x5 only by oral gavage) or RO6839921 and temozolomide treatment groups with 8 mice per group (Figure S1), and monitored by IVIS imaging for tumour growth. Baseline bioluminescence were measured prior to initiation of treatment on Day 1, and then during treatment on Days 8 (BLI1) and 15 (BLI2) (Figure S1). Twenty-four hours after the end of treatment, 3 mice per group were sacrificed for PK and PD analyses, and the remaining 5 mice per group were monitored for survival to humane endpoint (Figure S1). Animals were monitored 2-3 times weekly and euthanized humanely just before showing signs of illness or suffering such as abdominal dilatation, paraplegia, dehydration, or severe weight loss. Increase in lifespan (ILS) was calculated as a percentage based on both median and mean survival to humane endpoint, as previously described (4).

## *Detailed methods of pharmacokinetic analyses*

Chromatographic separation of idasanutlin was achieved using a Prominence HPLC (Shimadzu, Kyoto, Japan) with a Kinetex C18 50mm x 4.6mm 2.6µm and a SecurityGuard cartridge C18 3mm guard column (Phenomenex, California, USA) maintained at 30°C. Optimized HPLC conditions are given in Table S1A**.** An API4000 triple quadrupole LC-MS/MS (Applied Biosystems, California, USA) was used for analysis with electrospray ionization performed in positive ion mode. Optimized mass spectrometry conditions are given in Table S1B.

Tumour samples were prepared by addition of 3x tumour weight of water containing 0.2% dichlorvos (Sigma, UK) followed by homogenization using a hand-held Ultratorax in an ice jacket for 10 seconds. For sample analysis, 20µL plasma or tumour homogenate was used, to which 480µL ethyl acetate was added. The resulting precipitate was vortexed for 10 seconds followed by centrifugation at 4°C for 5min at 5000rcf. 300µL supernatant was then removed and dried down to residue under a steady stream of nitrogen gas. Samples were re-constituted in 150µL mobile phase before an injection of 10µL for analysis. Both neat and 1 in 100 fold dilutions were performed on all samples. A standard curve in matched matrix (blank plasma/blank tumour) was prepared fresh each analysis day.

Quality control samples were prepared in matched matrix at the concentrations 800ng/mL, 80ng/mL and 8ng/mL idasanutlin. The analytical method was validated (see below), with the following parameters determined: matrix effect, lower limit of quantification (LLOQ), linearity and range, intra- and inter-day precision and accuracy. Acceptance criteria for all analytical data were that all QC's had a precision and accuracy of 15% or better.

## *Analytical validation of idasanutlin detection*

No matrix effect was observed in 3 different batches of blank plasma and 2 separate blank tumour samples. The linear range of the assay was determined over 9 concentration points to be 3.9ng/mL to 1000ng/mL with a linear correlation of >0.993. The limit of quantitation was defined at 3.9ng/mL with a precision of <5% and an accuracy of within 15% of target concentration.

Intra assay variation was determined at 4%, 5% and 1% for the quality controls at 8, 80 and 800ng/mL respectively. Inter assay variation was determined at 5%, 5% and 3% for the quality controls at 8, 80 and 800ng/mL respectively.

# Supplementary Tables

**Table S1**

**A. HPLC gradient conditions**

| Minutes | 0.1% aqueous formic acid (%) | 0.1% formic acid in acetonitrile (%) |
|---|---|---|
| 0 | 30 | 70 |
| 1 | 30 | 70 |
| 2 | 0 | 100 |
| 3 | 0 | 100 |
| 4 | 30 | 70 |
| 5 | 30 | 70 |

**B. Mass spectrometry conditions**

| | |
|---|---|
| Precursor ion | 616.2 |
| Product ion | 421.1 |
| Curtain gas | 20 |
| Ion source gas 1 | 60 |
| Ion source gas 1 | 60 |
| Ionspray voltage | 5500V |
| Ionization temperature | 400°C |
| Collision gas | 6 |
| Dwell time | 150ms |
| Declustering potential | 171V |
| Entrance potential | 10V |
| Collision energy | 37eV |
| Collision cell exit potential | 12V |

## Supplementary Figures

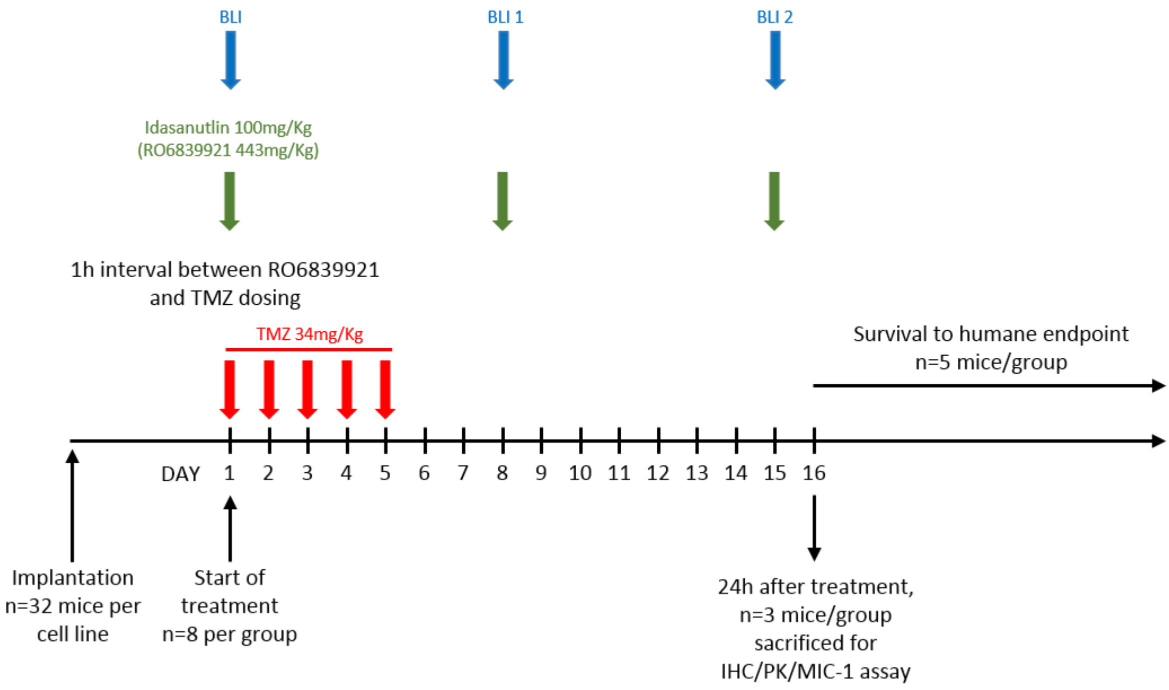


**Figure S1. Experimental schedule of efficacy study in SHSY5Y-Luc and NB1691-Luc orthotopic models.** The schedule tested represents one treatment cycle over 3 weeks. RG7775 was given IV on days 1, 8 and 15 of treatment, and temozolomide was given PO on days 1-5 of treatment. The dose and schedule of temozolomide used *in vivo* is equivalent to the clinical dose (100mg/m$^2$) of temozolomide, when used in combination in patients with neuroblastoma, and consistent with the currently used BEACON Trial (NCT02308527) dosing schedule. Treatment was started 19 days after implantation.

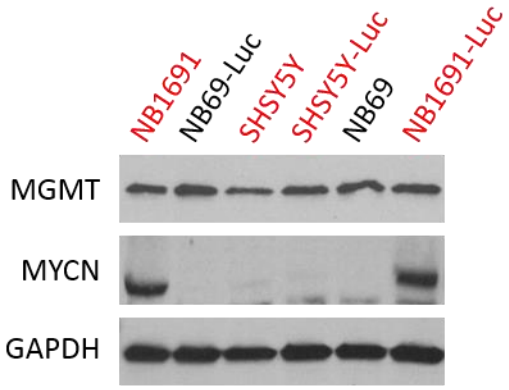


**Figure S2. MGMT levels in parental and luciferase cell lines.** Western analysis showing comparable levels of MGMT expression in parental and luciferase-tagged neuroblastoma cell lines. Specifically NB1691, NB1691-Luc, SHSY5Y and SHSY5Y-Luc are highlighted in red. MGMT antibody was used at 1:1000 (MAB16200, Merck Millipore).

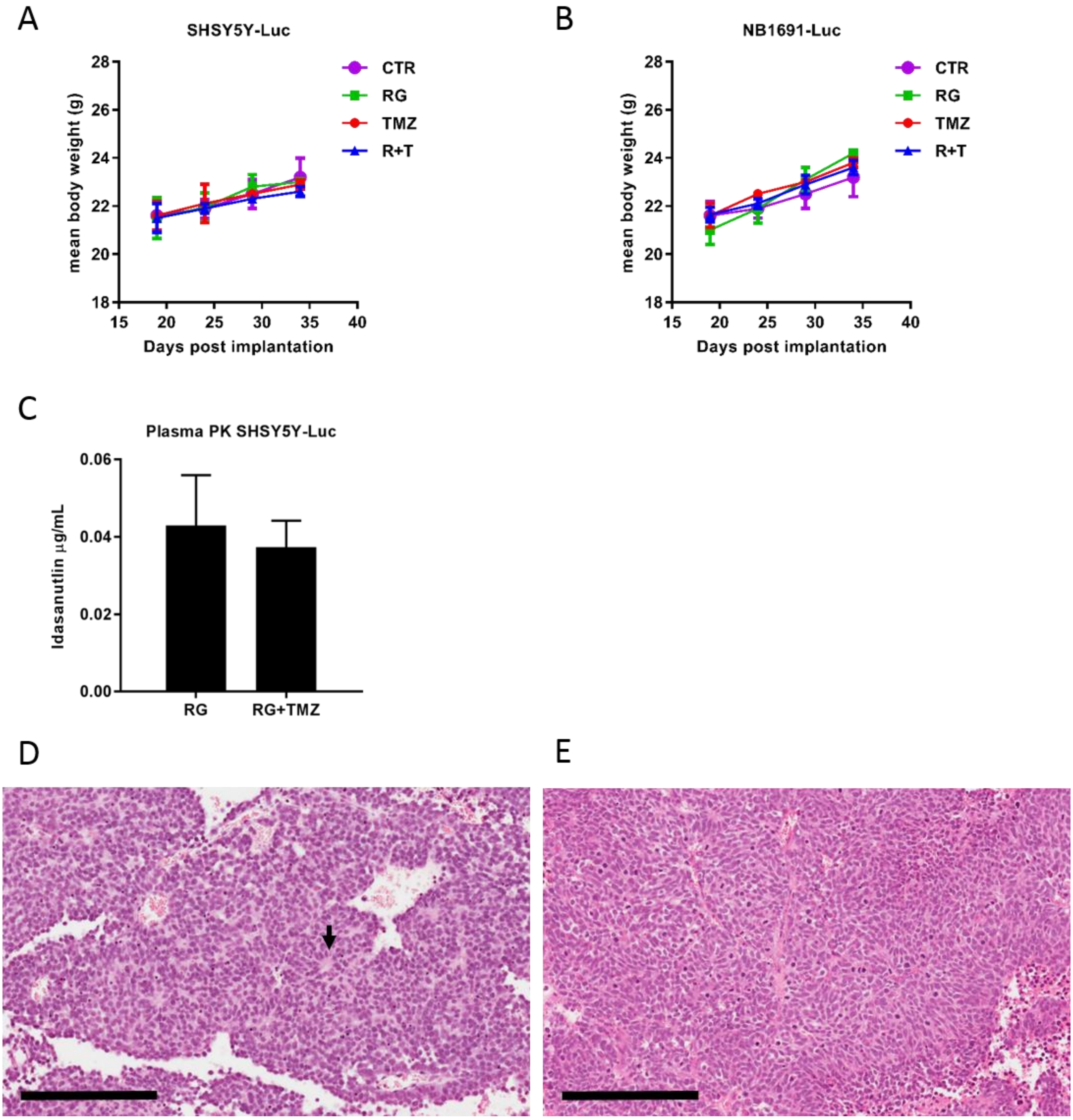


**Figure S3.** Mean weight of **(A)** SHSY5Y-Luc and **(B)** NB1691-Luc tumour bearing mice during duration of treatment n=8/group. CTR, control; RG, RO6839921; TMZ, temozolomide; R+T, RO6839921 and temozolomide. **(C)** PK analysis of active idasanutlin levels using LC-MS in plasma harvested 24h after treatment from SHSY5Y-Luc tumour bearing mice treated with a single dose of RO6839921 (RG) or RO6839921 and temozolomide (RG+TMZ) in combination. Hematoxylin and eosin staining of **(D)** SHSY5Y-Luc and **(E)** NB1691-Luc orthotopic tumours. Tumours are histologically comparable with primary human neuroblastoma tumours, indicating poorly differentiated neuroblastoma and a high mitosis-karyorrhexis index (INPC classification). Arrow indicates a typical Homer-Wright neuroblastoma rosette. Scale bar = 200 μm.

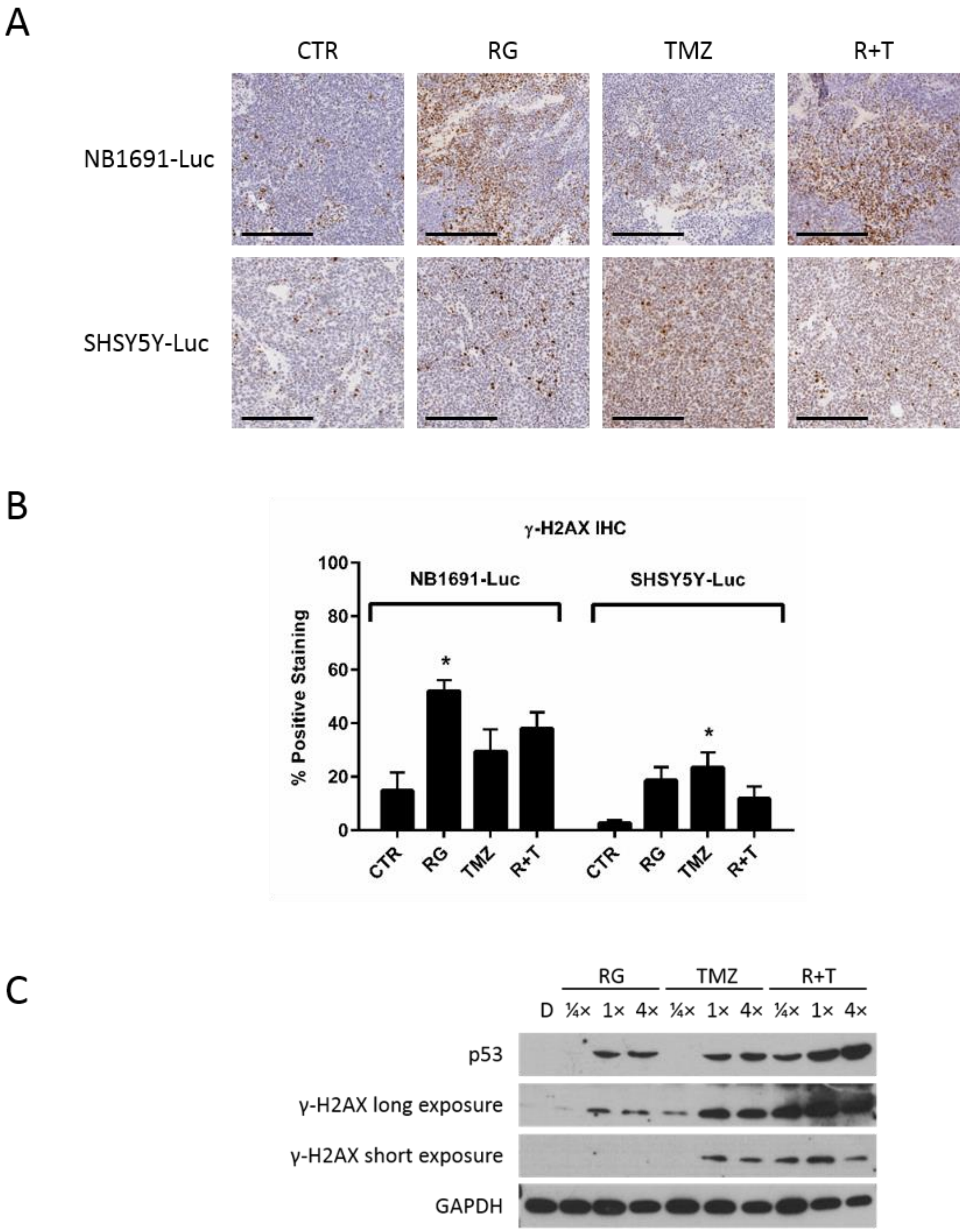


**Figure S4. A)** Representative images captured using the Aperio FL Digital Pathology Slide Scanner of γ-H2AX stained (20E3; Cell Signaling; 1:500) 5µm thick formalin-fixed, paraffin embedded NB1691-Luc and SHSY5Y-Luc tumour sections. Scale bar = 200 µm. B) Graphical representation of quantification of % positive γ-H2AX in NB1691-Luc and SHSY5Y-Luc orthotopic tumours. n=3 mice per group. All data are shown as the mean and error bars represent SEM. CTR, control; RG, RO6839921; TMZ, temozolomide; R+T, RO6839921 and temozolomide. Statistically significant differences were determined by one-way ANOVA with Bonferroni post-hoc tests and paired testing versus control. $P \leq 0.05$ (*). **C)** Western analysis of p53 and γ-H2AX expression in SHSY5Y cells treated *in vitro* with idasanutlin alone (RG), temozolomide alone (TMZ) or idasantlin and temozolomide in combination (R+T) at 0.25×, 1× and 4× their respective 72h $GI_{50}$ concentrations for 24 hours. γ-H2AX antibody (clone JBW301; Merck, 1:1000).

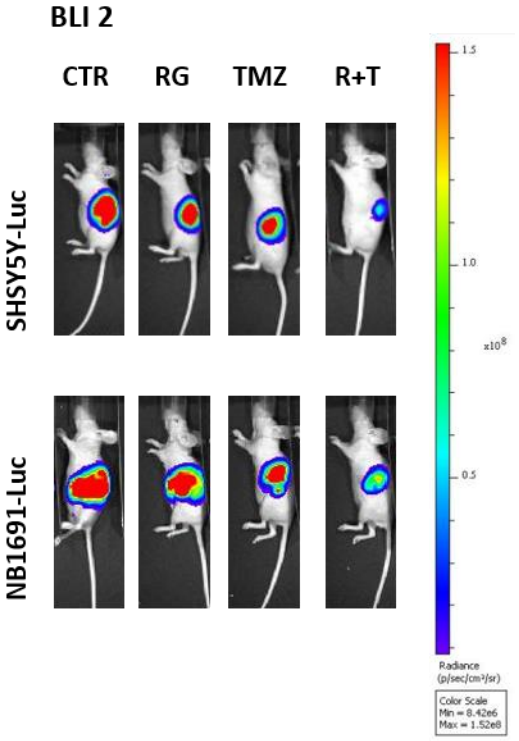


**Figure S5.** Bioluminescence emission of untreated (CTR) and treated SHSY5Y-Luc and NB1691-Luc orthotopic tumour bearing mice on Day 15 (BLI 2) of treatment with RO6839921 alone (RG), temozolomide alone (TMZ) or RO6839921 and temozolomide in combination (R+T).

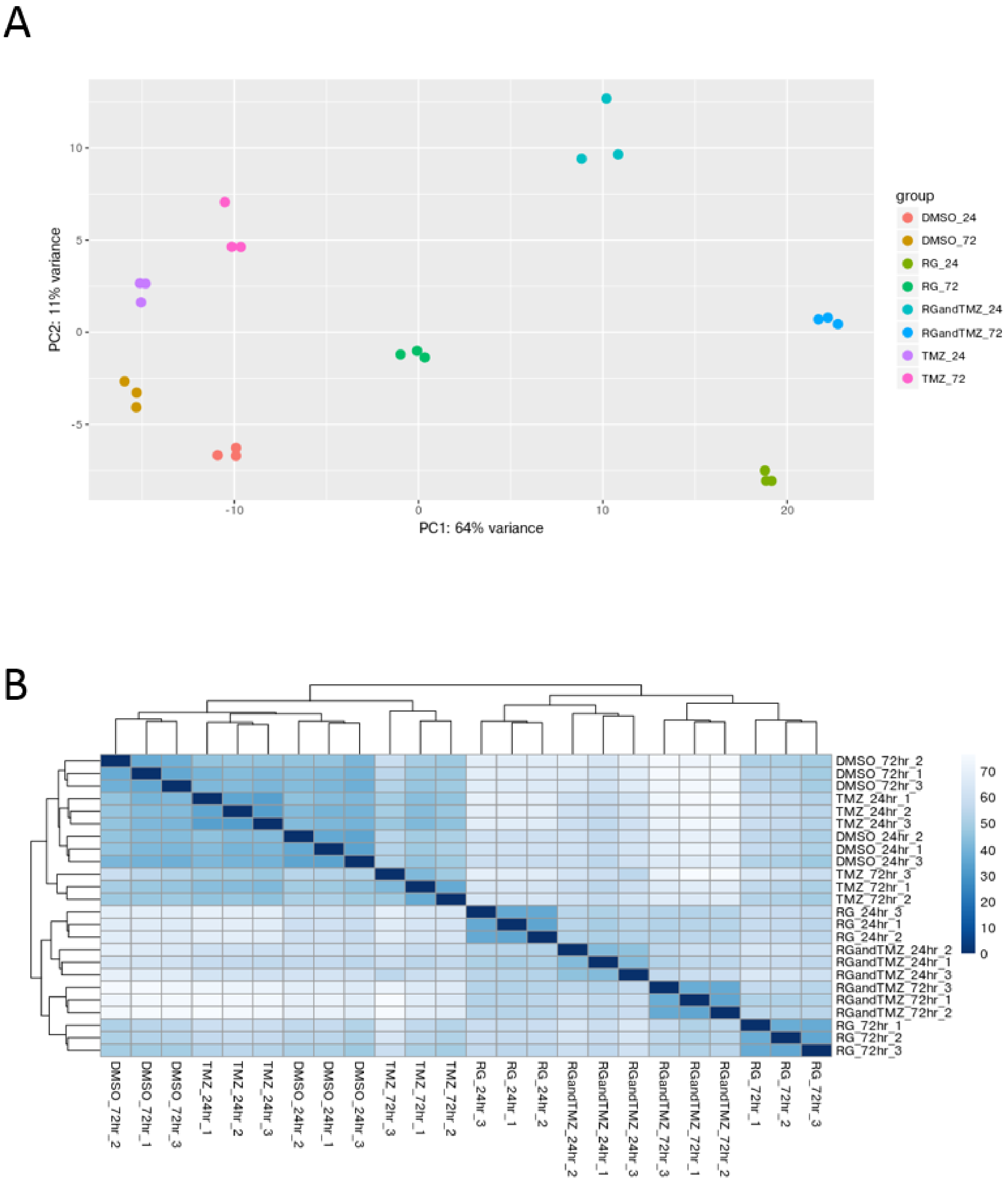


**Figure S6. Similarity between RNA-Seq samples. A)** PCA plot and **B)** Euclidean distance heatmap of the 24 NB1691 RNA-Seq samples, representing 8 conditions in triplicate (DMSO, idasanutlin (RG), temozolomide (TMZ), idasanutlin and temozolomide (RGandTMZ), at 24h and 72h).

A

| | GO | *P* value |
|---|---|---|
| **Up** | GO:0043065~positive regulation of apoptotic process | <0.01 |
| | GO:0006977~DNA damage response, signal transduction by p53 class mediator resulting in cell cycle arrest | <0.05 |
| | GO:0035556~intracellular signal transduction | <0.05 |
| **Down** | GO:0051301~cell division | <0.001 |
| | GO:0006260~DNA replication | <0.001 |
| | GO:0007067~mitotic nuclear division | <0.001 |

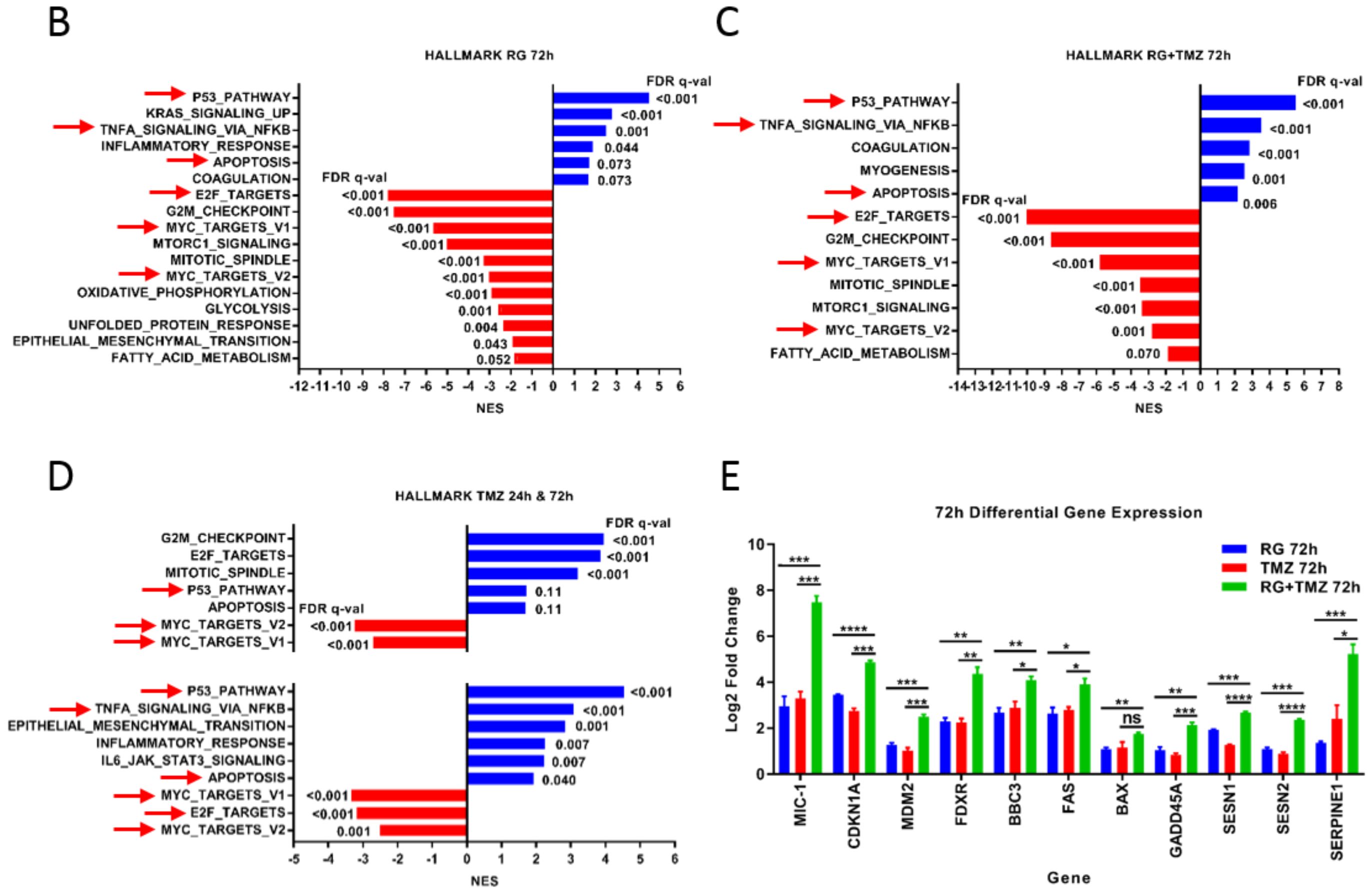


**Figure S7. Gene ontology and gene set enrichment analyses of NB1691 RNA-Seq samples. A)** Top 3 up- and downregulated GO of NB1691 cells treated with idasanutlin and temozolomide in combination at 72h from DAVID. *P* value = Benjamini FDR adjusted *P* value. Enriched gene sets from GSEA with MSigDB Hallmarks of NB1691 cells treated with **B)** idasanutlin for 72h, **C)** idasanutlin and temozolomide for 72h and **D)** temozolomide for 24h (top segment) and 72h (bottom segment). Red arrows indicate gene sets common to all treatment conditions. **E)** Graph showing the Log2 fold change in expression relative to DMSO control of p53 regulated genes of interest in NB1691 cells in response to idasanutlin and temozolomide alone and in combination for 72h. Statistical significance was determined using unpaired t-tests $P \leq 0.05$ (*); 0.01 (**); 0.001 (***); 0.0001 (****); ns, not significant. RG, idasanutlin; TMZ, temozolomide; RG+TMZ, idasanutlin and temozolomide.

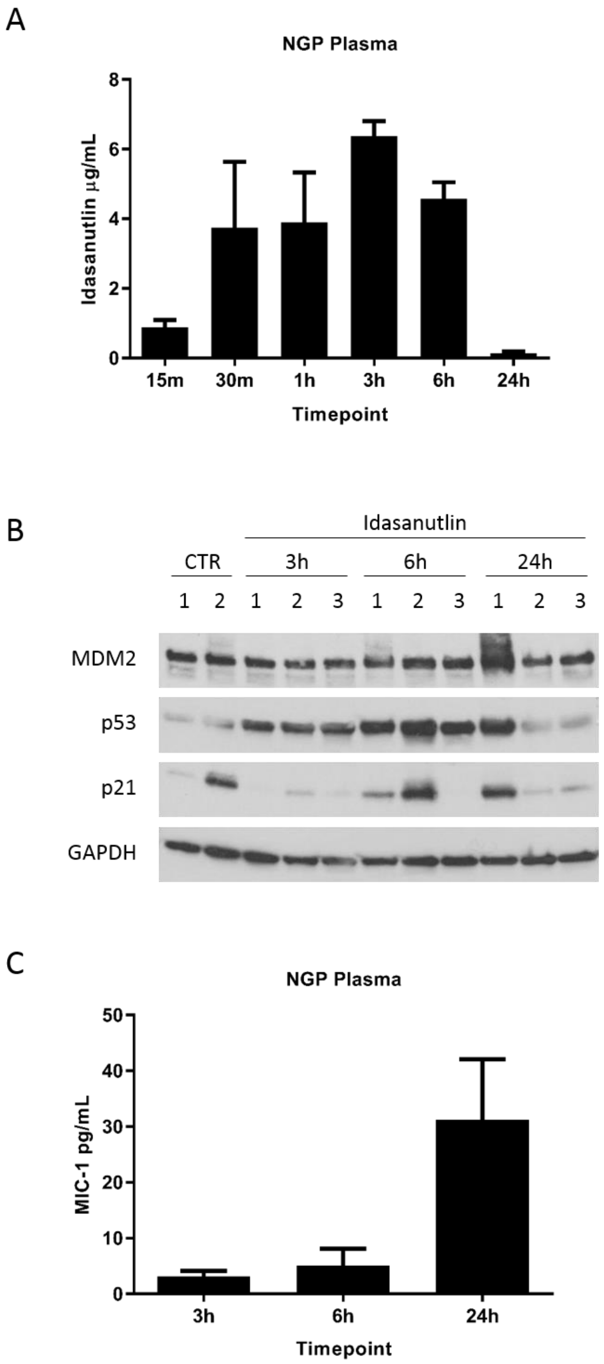


**Figure S8. PK and PD analysis of orally formulated idasanutlin in a *TP53* wt subcutaneous xenograft model of neuroblastoma.** Female CD1 nude mice were implanted subcutaneously with 1 × $10^7$ *TP53* wt, *MDM2* and *MYCN* amplified NGP neuroblastoma cells. **A)** PK analysis of idasanutlin levels using LC-MS in plasma samples harvested at the indicated time points from tumour bearing mice treated with a single dose equivalent to 100mg/kg of active idasanutlin by oral gavage. PD profiling for **B)** induction of the p53 pathway using Western analysis of p53, p21, and MDM2 levels in tumours, and **C)** MIC-1 levels in plasma harvested at the indicated time points from subcutaneous NGP tumour bearing mice treated with a single dose of idasanutlin by oral gavage. n=3 mice per time point. CTR, control.

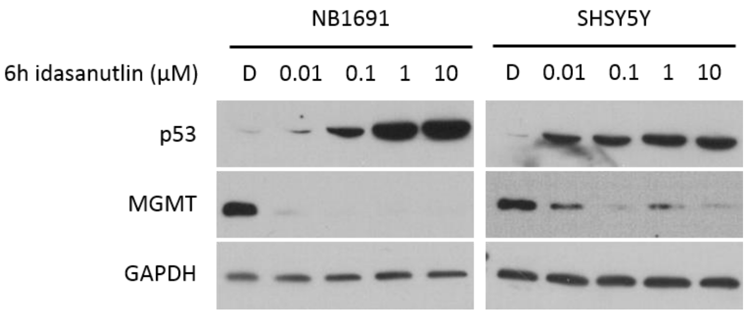


**Figure S9. MGMT expression in response to idasanutlin in *TP53* wt neuroblastoma cell lines.** Western analysis showing downregulation of MGMT expression in *TP53* wt NB1691 and SHSY5Y neuroblastoma cell lines treated for 6 hours with increasing concentrations of idasanutlin (10 nM, 100 nM, 1 μM, 10 μM) versus DMSO (D) control. MGMT antibody was used at 1:1000 (MAB16200, Merck Millipore).

## References


1. Pastorino F, Brignole C, Marimpietri D, Cilli M, Gambini C, Ribatti D, et al. Vascular damage and anti-angiogenic effects of tumor vessel-targeted liposomal chemotherapy. Cancer research. 2003;63:7400-9.
2. Pastorino F, Di Paolo D, Piccardi F, Nico B, Ribatti D, Daga A, et al. Enhanced antitumor efficacy of clinical-grade vasculature-targeted liposomal doxorubicin. Clin Cancer Res. 2008;14:7320-9.
3. Patterson DM, Shohet JM, Kim ES. Preclinical models of pediatric solid tumors (neuroblastoma) and their use in drug discovery. Current protocols in pharmacology / editorial board, SJ Enna. 2011;Chapter 14:Unit 14 7.
4. Higgins B, Glenn K, Walz A, Tovar C, Filipovic Z, Hussain S, et al. Preclinical Optimization of MDM2 Antagonist Scheduling for Cancer Treatment by Using a Model-Based Approach. Clin Cancer Res. 2014;20:3742-52.
